# The association between smoking and knee osteoarthritis in a cohort of Danish patients undergoing knee arthroscopy

**DOI:** 10.1186/s12891-019-2518-z

**Published:** 2019-04-01

**Authors:** Marianne Bakke Johnsen, Kenneth Pihl, Nis Nissen, Rasmus Reinholdt Sørensen, Uffe Jørgensen, Martin Englund, Jonas Bloch Thorlund

**Affiliations:** 1 Institute of Clinical Medicine, University of Oslo, Oslo, Norway; 2 Research and Communication Unit for Musculoskeletal Health, Oslo University Hospital, Building 37B, PO Box 4956, N-0424 Nydalen, Oslo Norway; 3 Research Unit for Musculoskeletal Function and Physiotherapy, Department of Sports Science and Clinical Biomechanics, University of Southern Denmark, Odense, Denmark; 4 Lillebaelt Hospital, Kolding, Denmark; 5 Department of Orthopaedic Surgery, Lillebaelt Hospital, Vejle, Denmark; 6 Odense University Hospital, Odense, Denmark; 7 Clinical Epidemiology Unit, Orthopedics, Department of Clinical Sciences Lund, Faculty of Medicine, Lund University, Lund, Sweden

**Keywords:** Smoking, Knee osteoarthritis, Meniscal tear, Knee arthroscopy, Risk factor

## Abstract

**Background:**

It has been suggested that smoking is associated with reduced risk of knee osteoarthritis (OA). However, supplementary studies are needed to further investigate any such potential association. Thus, our aim was to examine the relationship between smoking and early or more established knee OA in a cohort of relatively young patients with meniscal tears.

**Methods:**

This cross-sectional study included 620 participants from the Knee Arthroscopy Cohort Southern Denmark (KACS) undergoing knee arthroscopy for a meniscal tear (mean age 49.2 (18.0–76.8) years). Recruitment of patients was performed between February 1, 2013, and January 31, 2015, at four different hospitals in Denmark. We defined early or more established knee OA as the combination of patient-reported frequent knee pain, degenerative meniscal tissue and presence of cartilage defects assessed by the operating surgeons. The relationship between smoking status and knee OA was examined by risk ratio (RR) with a 95% confidence interval (CI), estimated from logistic regression adjusted for age, sex, BMI, education, work status and level of physical activity.

**Results:**

The prevalence of early or more established knee OA was 37.7% in current smokers and 45.0% in non-smokers. We found no statistically significant association between current smoking and knee OA (adjusted RR 1.09, 95% CI 0.91–1.30).

**Conclusions:**

This study found no relationship between current smoking and early or more established knee OA in a cohort of patients undergoing arthroscopic meniscal surgery. Thus, the inverse association between smoking and knee OA that has been suggested by previous studies was not confirmed.

## Background

An inverse association between smoking and knee osteoarthritis (OA) has been suggested to exist, meaning that smoking may be protective of OA. A recent meta-analysis reported that this inverse relationship seems to exist regardless of study design, and that the association is stronger in men compared to women [1]. However, not all studies have identified this relationship between smoking and knee OA [1, 2]. Currently, the exact mechanisms by which smoking might protect against OA are not clear, although a positive effect of nicotine on chondrocyte metabolism has been proposed by in vitro studies [3, 4]. On the contrary, studies have demostrated inconsistent results when studying the effect of smoking in patients by assessing articular cartilage volume using MRI [5, 6]. As any causal relationship between smoking and knee OA is still speculative, there is a need for studies to further elucidate this association. In previous studies, mainly radiographic definitions of knee OA or total knee replacement have been used to define the presence of severe knee OA [1]. Individuals with degenerative meniscal tears often exhibit early signs of knee OA and are considered to have a higher risk of developing the disease [7] and may therefore be a useful population to study the relationship between early degenerative knee changes and smoking. Therefore, the purpose of this study was to examine whether there is a relationship between smoking and early or more established knee OA in a cohort of patients with meniscal tears.

## Methods

This cross-sectional study included participants from the Knee Arthroscopy Cohort Southern Denmark (KACS), which is a longitudinal cohort following patients undergoing knee arthroscopy for a meniscal tear [8]. The KACS cohort recruitment and inclusion criteria have previously been described in more detail in a protocol paper by Thorlund et al. [8], and by Pihl et al. [9]. In short, all patients were recruited between February 1, 2013 and January 31, 2015. Patients who were 18 years and older, and who were assigned for knee arthroscopy based on suspicion of a medial and/or lateral meniscus tear were included. In addition, patients had to understand and be able to read Danish and have an email address [8, 9]. Further, exclusion criteria were: self-reported planned or previous anterior or posterior cruciate ligament (ACL or PCL) reconstruction in either knee, fracture(s) to either leg within the last six months at time of recruitment or not being able to answer the questionnaire due to mental impairment [8, 9]. The Regional Scientific Ethics Committee of Southern Denmark exempted the study for ethical approval, as the study only utilizes questionnaire and register data. Still, all patients who participated in the study signed a written informed consent form [8].

### Exposure definition

Main exposure was smoking status, and this data was collected through an email-based questionnaire prior to the arthroscopic surgery (median of 7 days prior to surgery, IQR 3–10 days), as reported previously [9]. Participants were asked: “Do you smoke?”. The reply options were: “no”, “no, stopped within the last six months”, “yes, rarely”, “yes, 1-5 cigarettes a day”, “yes, 6-10 cigarettes a day”, “yes, 11-20 cigarettes a day”, “yes, more than 20 cigarettes a day”, “yes, smoke pipe”. Non-smokers were defined as those reporting “no” or “no, stopped within the last 6 months”. Never and former smokers were both categorized as non-smokers (*n* = 482) due to the low number of former smokers (*n* = 12). Current smokers were defined as those who reported smoking cigarettes (regardless of number of cigarettes) or a pipe. Surgeons were not aware of what the patients had replied in the email-based questionnaire.

### Covariates

Relevant covariates that were available from the email-based questionnaire were age, sex, highest completed level of education, work status, body mass index (BMI; calculated from baseline self-reported height and weight) and physical activity in leisure time assessed using a previously validated question [10, 11]. Participants were asked “What education do you have?”. The reply options were: “primary school”, “high school”, “vocational education”, “short-cycle higher education”, “medium higher education”, “bachelor degree”, “master”, “PhD” and “other”. We categorized education into primary school, high school/vocational training (including short-cycle higher education), bachelor level (including medium higher education) or master/PhD/other. Work status was assessed by asking “Are you currently working?”, with reply options: “full time”, “part time”, “retired”, “unemployed”, “student” and “other”. We categorized work status into employed (fulltime/part time/student/other) or unemployed (retired/unemployed). Physical activity in leisure time was defined as low (hardly any activity, or light, non-strenuous activity once a week), medium (regular activity at least once a week) and high (regular, strenuous activity more than once a week).

### Knee symptoms

Additionally, patients answered the Knee injury and Osteoarthritis Outcome Score (KOOS) prior to surgery. The KOOS questionnaire comprises 5 individual subscales: pain, symptoms, activities of daily living (ADL), sport and recreation function (Sport/Rec) and knee related quality of life (QoL), as described previously by Roos et al. [12]. The range of each subscale is from 0 to 100 points, with 0 indicating extreme knee problems and 100 indicating no knee problems. The KOOS has shown to be a valid, reliable and responsive patient-reported outcome measure for patients with several types of knee injuries and knee OA [13].

### Knee pathology at arthroscopy

At the time of surgery, the operating surgeon recorded information about the quality of the meniscal tissue (i.e. non-degenerative, degenerative or undetermined) and degree of cartilage defects in each knee joint compartment. We classified cartilage defects as described previously in the KACS cohort [9], using the International Cartilage Repair Society (ICRS) grading system (grade 0 to 4) [14], where grade 0 represents normal cartilage and 4 represents severe cartilage lesions.

### Outcome definition

We defined early or more established knee OA, as described by Pihl et al. [9], based on the combination of frequent knee pain, using only one item from the KOOS pain subscale (response alternatives “daily” or “always”), degenerative meniscal tissue (assessed by the surgeon at time of surgery) and cartilage defect (i.e. ICRS grade 1 in at least two knee joint compartments or at least ICRS grade 2 in one compartment). The outcome was categorized as a binary variable; early or more established knee OA versus no knee OA.

### Statistical analysis

Descriptive cohort characteristics are presented as means and standard deviations (SD) or numbers and percentages. We used a Chi squared test or an unpaired t-test, as appropriate, to compare descriptive characteristics. The relationship between smoking and knee OA was calculated by logistic regression with estimation of the risk ratio (RR) with a 95% confidence interval (CI), using the method described by Norton et al. [15]. The full model was adjusted for age, sex, BMI, education, work status and level of physical activity. In a sensitivity analysis, we excluded adjustment for BMI due to the potential of collider bias [16]. We tested for interaction between smoking and sex based on previous results [1]. We compared the model with and without the interaction term using the likelihood ratio test. Finally, we checked the residuals and leverage of the model. We used Stata/SE 14.1 to perform all statistical analyses. *P*-value less than 0.05 was considered to be statistically significant.

## Results

**Fig. 1:**
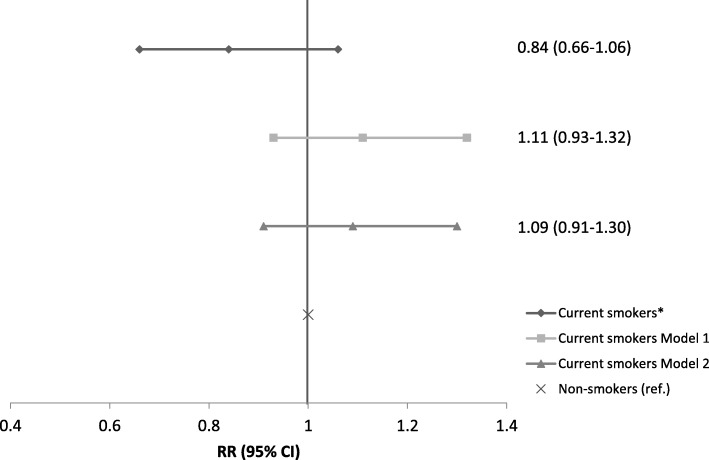
Association between smoking and early or more established knee OA. *Crude: logistic regression with estimation of risk ratio (RR) and 95% CI. Model 1: logistic regression with RR and 95% CI, adjusted for age, sex and BMI. Model 2: logistic regression with RR and 95% CI, adjusted for age, sex, BMI, highest completed level of education, work status and the level of leisure time physical activity

The study sample comprised 620 patients. Of these, 22.3% were current smokers and 77.7% were non-smokers. Current smokers compared with non-smokers were younger at age, had somewhat lower BMI and fewer had completed higher education (Table 1). Moreover, current smokers reported worse knee symptoms and function, according to the KOOS score, compared to non-smokers (Table 2). The prevalence of early or more established knee OA (as defined) was 37.7% (52 of 138) in current smokers and 45.0% (217 of 482) in non-smokers. Of those with knee OA (*n* = 269), 37.2% had ICRS grade 1 in at least two knee joint compartments or ICRS grade 2 in one compartment, while 62.8% had ICRS grade 3 or 4 in at least one knee compartment. There was no statistically significant relationship between current smoking and knee OA in any of the models (Fig. 1). The results did not change substantially after excluding adjustment for BMI (RR 1.05, 95% CI 0.88–1.27), nor did we find any interaction between smoking and sex (p interaction term = 0.76).

**Table 1:** Patient characteristics

Variables	Current smokers (*n* = 138)	Non-smokers (*n* = 482)	*P*-value^a^
Age, years (SD)	45.0 (12.2)	50.4 (12.9)	< 0.001
BMI, kg/m^2^ (SD)	26.7 (4.4)	27.4 (4.4)	0.08
Female sex, n (%)	54 (39.1)	213 (44.2)	0.29
Education, n (%)			
Primary school	28 (20.3)	73 (15.2)	
High school/vocational training	68 (49.3)	247 (51.2)	
Bachelor level	40 (29.0)	119 (24.7)	
Master/PhD/other	2 (1.5)	43 (8.9)	0.01
Work status, n(%)			
Unemployed	27 (19.6)	119 (24.7)	
Employed (or self-employed)	111 (80.4)	363 (75.3)	0.21
Physical activity, n (%)			
Low	35 (25.3)	92 (19.1)	
Medium	51 (37.0)	190 (39.4)	
High	52 (37.7)	200 (41.5)	0.27

*BMI* body mass index

^a^Chi squared test for categorical variables and an unpaired t-test for continuous variables

**Table 2:** Description of patient symptoms and knee pathology

Variables	Current smokers (*n* = 138)	Non-smokers (*n* = 482)	*P*-value^d^
Frequency of knee pain^a^, n (%)			
Never	3 (2.2)	6 (1.2)	
Monthly	7 (5.1)	21 (4.4)	
Weekly	15 (10.9)	55 (11.4)	
Daily	88 (63.8)	313 (64.9)	
Always	25 (18.1)	87 (18.1)	0.94
KOOS scores^b^, mean (95% CI)			
Pain	52.3 (49.2–55.3)	55.6 (54.0–57.3)	0.06
Symptoms	56.6 (53.5–59.7)	60.9 (59.2–62.5)	0.02
ADL	60.0 (56.8–63.1)	64.8 (63.0–66.5)	0.01
Sport/Rec	22.5 (19.2–25.9)	27.3 (25.3–29.3)	0.02
QoL	37.9 (35.3–40.5)	42.7 (41.3–44.0)	0.001
Meniscal tissue quality, n (%)			
Non-degenerative	65 (47.1)	180 (37.3)	
Degenerative	69 (50.0)	286 (59.3)	
Undetermined	4 (2.9)	16 (3.3)	0.11
ICRS cartilage grade^c^, n (%)			
Medial compartment			
Grade 0	42 (30.4)	136 (28.2)	
Grade 1	38 (27.5)	108 (22.4)	
Grade 2	28 (20.3)	88 (18.3)	
Grade 3	28 (20.3)	111 (23.0)	
Grade 4	2 (1.5)	39 (8.1)	0.06
Lateral compartment			
Grade 0	66 (47.8)	197 (40.9)	
Grade 1	48 (34.8)	158 (32.8)	
Grade 2	15 (10.9)	77 (16.0)	
Grade 3	9 (6.5)	36 (7.5)	
Grade 4	0	14 (2.9)	0.11
Patellofemoral compartment			
Grade 0	54 (39.1)	174 (36.1)	
Grade 1	45 (32.6)	122 (25.3)	
Grade 2	19 (13.8)	85 (17.6)	
Grade 3	16 (11.6)	73 (15.2)	
Grade 4	4 (2.9)	28 (5.8)	0.20

^a^Single item from the KOOS pain subscale

^b^Score ranging from 0 to 100, with 0 indicating extreme problems and 100 indicating no problems

^c^ICRS grad = International Cartilage Repair Society grading system. Grade 0 representing normal cartilage and 4 representing very severe cartilage lesion

^d^Chi squared test for categorical variables and an unpaired t-test for continuous variables

*ADL* activities of daily living, *Sport/Rec* sport and recreational activities, *QoL* quality of life

## Discussion

In this study, we found no evidence of an association between current smoking and early or more established knee OA. Thus, the potential inverse relationship between smoking and knee OA could not be confirmed.

In contrast to the meta-analysis that reported an inverse relationship between smoking and knee OA to be independent of study design [1], a previous study including individuals from the Osteoarthritis Initiative (OAI), with knee OA based on Kellgren-Lawrence (KL) grade ≥ 2, found an association between smoking and knee symptoms and structural disease status that was dependent on study design [2]. The cross-sectional analysis indicated worse knee function but greater joint space width (JSW) in current smokers compared to never smokers. However, there was no difference in symptom changes or JSW between smokers and never smokers in the longitudinal analysis. The authors believed the longitudinal analysis to be more robust and concluded that the differences found in the cross-sectional analysis could be due to residual confounding [2]. In the Clearwater Osteoarthritis Study, a prospective study of 2505 individuals, there was an indication of a protective effect of current smoking on the risk of OA in the hand, hip, knee and foot in unadjusted analyses [17]. However, after the adjustment of covariates such as age, gender, BMI and physical activity [17] the relationship disappeared; this was also observed in the present study.

Similar to participants in the Osteoarthritis Initiative [2], smokers in the current study reported worse knee symptoms and function compared to non-smokers according to the subscales of KOOS. However, the difference was small and probably not clinically important as differences around 10 points are usually considered to constitute a minimal clinically relevant difference in KOOS subscales [13]. We observed no statistically significant difference between smokers and non-smokers in self-reported knee pain. Smoking has been suggested to have an analgesic effect through the constituent of nicotine [18]. Nevertheless, smokers have also been found to have a higher risk of musculoskeletal pain compared to non-smokers [18]. Smokers also tend to report more intense pain than non-smokers when musculoskeletal pain is present [18]. In the current study, however, all participants were expected to have a certain degree of knee pain, regardless of smoking status, as all patients underwent surgery for a meniscal tear and knee pain.

One hypothesis is that smoking might decrease the risk of knee OA through a positive effect of nicotine on articular chondrocyte function [4, 6]. In a sample of 297 adults without knee OA or any history of knee injury from the Melbourne Collaborative Cohort Study, ever smoking was associated with an increase in tibial cartilage volume, when compared to never smoking, but not with the presence of cartilage defects [6]. Furthermore, the study reported a positive association, suggesting a dose-dependent relationship between the number of pack-years and cartilage volume [6]. We did not assess cartilage volume in our study. However, the operating surgeons in our study reported a greater proportion of degenerative meniscal tissue quality in non-smokers versus current smokers (59.3% vs 50.0%), though this was not statistically significant. Our crude analysis supported this descriptive finding, indicating a reduced prevalence of knee OA among current smokers compared to non-smokers. However, after adjustments, mainly for age, the direction of the association changed. A second hypothesis is that smoking might affect the risk of knee OA through its effect on BMI [16]. However, one former study showed that the indirect effect of smoking on hip and knee OA was small [19], as was indicated by the sensitivity analysis in the present study.

This study has important limitations. For instance, its cross-sectional design limits the ability to make causal inferences. We used self-reported smoking status as the main exposure, which is a crude measure of smoking exposure with risk of misclassification. Further, we categorized former smokers who had stopped smoking within the last 6 months as “non-smokers”. However, the number of former smokers was low (*n* = 12). A misclassification may have decreased any potential differences between current and non-smokers because of similarities in the smoking exposure of the two groups. Although surgeons were blinded to the patients’ responses to smoking status in the email-based questionnaire, patients may have made the surgeon aware of it during the consultation. However, at the time of surgery, the surgeons were not aware of any particular intention to study the association between smoking and cartilage status. Thus, it is unlikely that knowledge of the patient’s smoking status would have influenced their cartilage grading. Due to the small sample size we did not further stratify the main analysis, e.g. by OA severity, gender or age. However, as mentioned, there was no sign of statistical interaction between smoking and sex. We only investigated subjects selected for knee arthroscopy and it is unclear whether smoking status or other patient characteristics may have influenced this selection for surgery, thereby affecting our results. In addition, since all patients had a certain degree of knee pain and cartilage degeneration, we cannot disregard that this might have attenuated the potential association between smoking and knee OA. However, the proportion of smokers in this cohort (22%) is similar to the general Danish population [20]. To the best of our knowledge, this is the first study to investigate the association between smoking and early or more established knee OA, as currently defined, in a relatively young cohort of patients. Previous studies have primarily used KL ≥ 2 or total knee replacement as a measure of radiographic or severe knee OA and patients being substantially older. It has been shown that meniscal tears are associated with OA even in knees with KL grade 0, thus, degenerative meniscal tears are thought to be one of the key factors in the early development of knee OA [7].

## Conclusion

In conclusion, this study found no relationship between current smoking and early or more established knee OA in a cohort of patients undergoing knee arthroscopy for a meniscal tear. Meniscal tears, cartilage defects and knee pain may be important markers of knee OA and represent a different phenotype of knee OA than has previously been studied in relation to smoking.

## Abbreviations

ADL: Activities of daily living
BMI: Body mass index
CI: Confidence intervals
ICRS: International Cartilage Repair Society
IQR: Interquartile range
JSW: Joint space width
KACS: Knee Arthroscopy Cohort of Southern Denmark
KL: Kellgren-Lawrence
KOOS: Knee injury and Osteoarthritis Outcome Score
MRI: Magnetic resonance imaging
OA: Osteoarthritis
QoL: Quality of life
RR: Risk ratio
SD: Standard deviations
